# Conserved plant transcriptional responses to microgravity from two consecutive spaceflight experiments

**DOI:** 10.3389/fpls.2023.1308713

**Published:** 2024-01-08

**Authors:** Eric S. Land, James Sheppard, Colleen J. Doherty, Imara Y. Perera

**Affiliations:** ^1^ Department of Plant and Microbial Biology, North Carolina State University, Raleigh, NC, United States; ^2^ Department of Molecular and Structural Biochemistry, North Carolina State University, Raleigh, NC, United States

**Keywords:** transcriptional profiling, adaptation to spaceflight, Arabidopsis, microgravity (μ*g*), plants

## Abstract

**Introduction:**

Understanding how plants adapt to the space environment is essential, as plants will be a valuable component of long duration space missions. Several spaceflight experiments have focused on transcriptional profiling as a means of understanding plant adaptation to microgravity. However, there is limited overlap between results from different experiments. Differences in experimental conditions and hardware make it difficult to find a consistent response across experiments and to distinguish the primary effects of microgravity from other spaceflight effects.

**Methods:**

Plant Signaling (PS) and Plant RNA Regulation (PRR) were two separate spaceflight experiments conducted on the International Space Station utilizing the European Modular Cultivation System (EMCS). The EMCS provided a lighted environment for plant growth with centrifugal capabilities providing an onboard 1 *g* control.

**Results and discussion:**

An RNA-Seq analysis of shoot samples from PS and PRR revealed a significant overlap of genes differentially expressed in microgravity between the two experiments. Relative to onboard 1 *g* controls, genes involved in transcriptional regulation, shoot development, and response to auxin and light were upregulated in microgravity in both experiments. Conversely, genes involved in defense response, abiotic stress, Ca^++^ signaling, and cell wall modification were commonly downregulated in both datasets. The downregulation of stress responses in microgravity in these two experiments is interesting as these pathways have been previously observed as upregulated in spaceflight compared to ground controls. Similarly, we have observed many stress response genes to be upregulated in the 1 *g* onboard control compared to ground reference controls; however these genes were specifically downregulated in microgravity. In addition, we analyzed the sRNA landscape of the 1 *g* and microgravity (μ *g*) shoot samples from PRR. We identified three miRNAs (miR319c, miR398b, and miR8683) which were upregulated in microgravity, while several of their corresponding target genes were found to be downregulated in microgravity. Interestingly, the downregulated target genes are enriched in those encoding chloroplast-localized enzymes and proteins. These results uncover microgravity unique transcriptional changes and highlight the validity and importance of an onboard 1 *g* control.

## 1 Introduction

As an essential part of life support systems for long-duration space missions, plants supplement food as well as purify the air and water (Fu et al., 2016; De Micco et al., 2023). Future colonization efforts will require the ability to grow plants in unfamiliar environments. However, the space environment poses challenges to growing plants. In addition to the difficulties of providing essential requirements for plant growth (light, water, nutrients, etc.), growing plants in space requires the mitigation of unfavorable factors such as altered gas composition, lack of convective currents, and cosmic radiation.

It is important to understand the molecular mechanisms that regulate plant adaptations to space. Toward that goal, several spaceflight experiments have examined the global transcriptional changes of plants grown on the International Space Station (ISS). One caveat to these studies is that until recently, lighted habitats for plant experimentation were limited; for ease of handling and setup, several experiments were carried out in Biological Research In Canisters hardware (BRICs), where plants were grown in the dark. Another point of concern is that many spaceflight experiments have employed hardware-matched experiments on Earth as controls; however, it is difficult to replicate all of the stresses that plants may face on the ISS on the ground.

Further, while several transcriptional datasets have been generated (accessible through the NASA GeneLab data repository), differences in plant age, tissue type, and growth conditions confound efforts to separate primary responses to microgravity from other spaceflight related stress responses (Manzano et al., 2022; Olanrewaju et al., 2023). A recent meta-analysis by Barker et al. clearly illustrates some of these confounding effects (Barker et al., 2023). The authors showed that the analysis method (RNA-Seq vs. microarrays) was a principal factor leading to variability between datasets as was the hardware utilized for the spaceflight experiments. Nevertheless, focusing only on experiments conducted in BRIC hardware helped uncover common responses. Namely, oxidative stress, heat shock, cell wall dynamics, hypoxia, and ROS signaling were Gene Ontology annotations enriched in spaceflight compared to ground controls. A transcriptional profiling study of 12 day-old Arabidopsis seedlings grown in the Advanced Biological Research System (ABRS) hardware found organ-specific gene expression changes between leaves, hypocotyls, and roots (Paul et al., 2013). Although each tissue exhibited unique responses, cell wall remodeling, touch response, and pathogen responses were common themes among the genes differentially expressed in space.

Plant growth is highly responsive to external cues. Two critical cues –light and gravity– have profound influences on the direction and magnitude of plant growth. Plant roots grow down with the gravity vector (positive gravitropism), while the stems grow opposite to the gravity vector (negative gravitropism) and toward light (positive phototropism). On earth, light and gravity work in combination to direct the orientation of the primary axes of growth as well as root and shoot branching and leaf angle, enabling efficient nutrient acquisition and light capture. The relative contribution of each of these cues in regulating plant growth is difficult to disentangle on Earth, where gravity is a constant; the ISS provides an ideal platform to answer such fundamental questions.

The European Modular Cultivation System (EMCS) was one of the controlled environment plant growth habitats operational on the ISS for many years (Brinckmann, 1999; Kittang et al., 2014). In addition to providing lighting, regulated air circulation, and ethylene scrubbing, the EMCS was unique in that it consisted of two centrifuge rotors within a growth chamber. Rotor speeds could be varied to impart *g* levels ranging from 0 - 2 *g*. The EMCS, therefore, afforded researchers the ability to conduct a simultaneous 1 *g* control in space as well as to specifically query the effects of partial gravity.

Spaceflight experiments utilizing EMCS hardware have attempted to uncouple light and gravity stimuli (Millar et al., 2010; Vandenbrink et al., 2016). Some experiments have examined the transcriptional response of plants to partial *g* levels. We previously reported that a subset of genes differentially expressed in microgravity respond to incremental increases in *g* levels (Sheppard et al., 2021). Other researchers have compared differences in gene expression between Lunar or Martian gravity and onboard 1 *g* controls (Herranz et al., 2019; Villacampa et al., 2021). These types of experiments can inform us on plant responses to extraterrestrial habitats and will be important for aiding with long term colonization efforts.

In this study, we compare the transcriptional response of Arabidopsis shoots from two independent spaceflight experiments conducted on the ISS. In both experiments, Arabidopsis seedlings were grown for 5-6 days in the EMCS. The results revealed considerable overlap between the two experiments despite differences in the experimental setup. Furthermore, onboard 1 *g* controls facilitated the identification of microgravity-specific differences in gene expression.

## 2 Materials and methods

### 2.1 Preflight ground testing

Prior to flight build, optimization was carried out at North Carolina State University with a focus on seed stock viability, experimental protocols, and downstream sample recovery and processing. *Arabidopsis thaliana* Col-0 seed stocks were carefully screened to obtain an average germination of ≥99%, and dissection and processing protocols were developed to recover ≥1μg RNA from ≤3mg input tissue with a high degree of RNA integrity. Seed storage tests were carried out to ensure the viability of sterilized seeds for at least a 6 month period. Preflight testing for Plant Signaling (PS) included a Schedule Test and Operations Verification Test (OVT) at the Norwegian User Support and Operations Center (N-USOC, Trondheim, Norway), in an Engineering Reference Module (ERM-1). Similarly, ahead of the Plant RNA Regulation (PRR) spaceflight, an Experiment Verification Test (EVT) at NASA Ames Research Center (ARC) in an EMCS ground facility (ERM-2), and an Operations Verification Test (OVT) in the EMCS Engineering Reference Module (ERM-1) at N-USOC were carried out. All preflight and flight preparations for seed, media, and hardware assembly were carried out at NASA ARC.

### 2.2 Flight experiment preparation

PS and PRR were carried out in the EMCS using the experimental unique equipment (EUE)s which are the TROPI-like seed cassettes previously described (Kiss et al., 2009; Vandenbrink and Kiss, 2019). Whatman #3 blotter paper and black-gridded PES membrane (PALL life Sciences Cat. No. 65561) were cut to fit the seed cassettes. Blotter papers were pre-soaked in 0.5X MS media, pH 5.7 (without sucrose) and allowed to dry. Blotter papers and membranes were sterilized by autoclaving. Seeds were surface sterilized and allowed to dry for 2 hours. Healthy viable seeds were selected under a dissecting microscope and attached to the gridded membrane using guar gum. Seeds were positioned with micropyles pointing down (away from lights) with 27 seeds/membrane. Gridded seeded membranes were attached to prepared blotters with guar gum and fixed to cassette base plates. Cassette covers were installed and sealed with foil tape (3M). A total of 80 cassettes were prepared for each experiment. Seed cassettes were assembled for flight at NASA ARC and loaded into experimental containers (ECs) approximately 4 weeks prior to the scheduled flight.

### 2.3 Spaceflight

**Figure 1:**
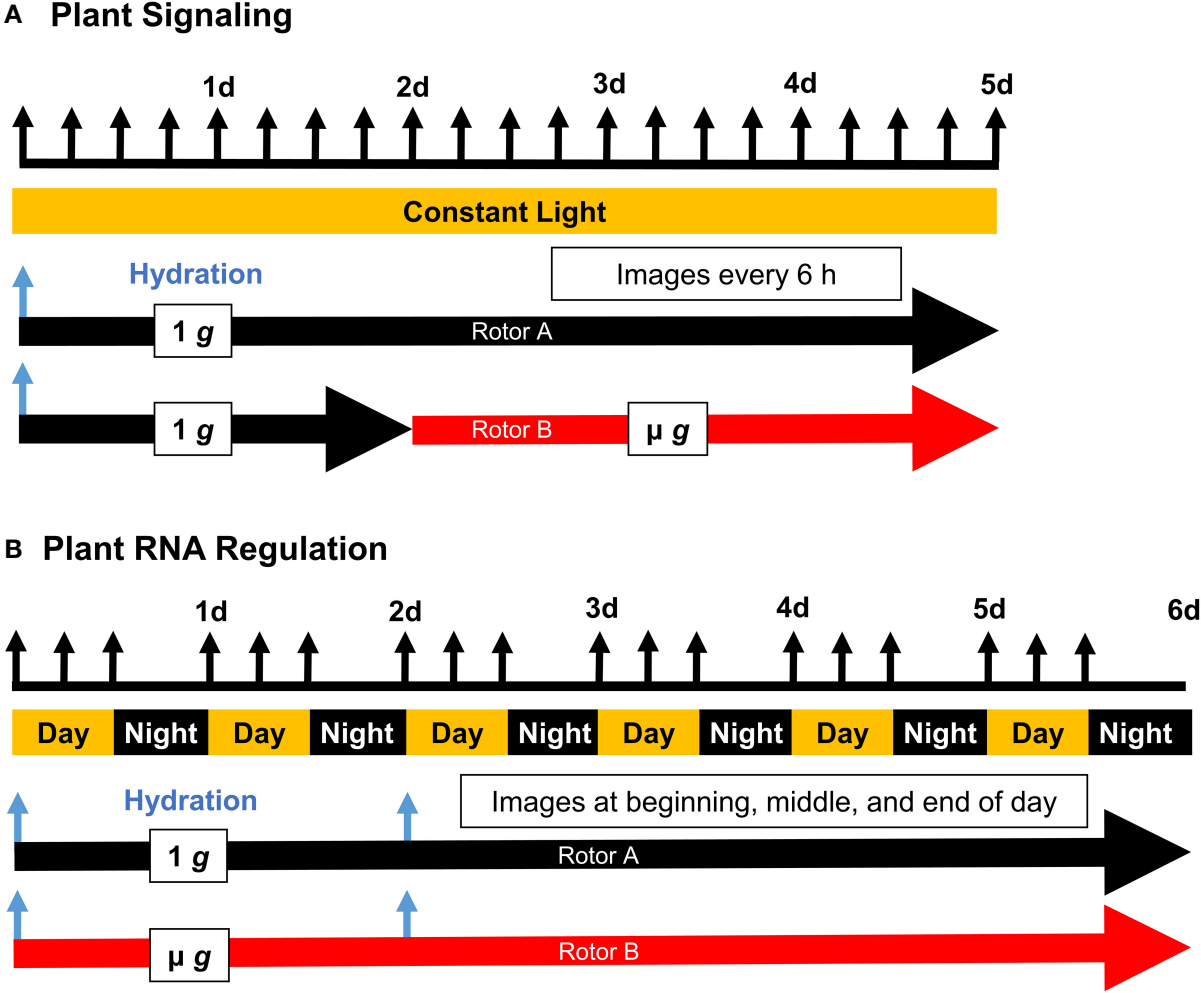
Experimental Timelines of Plant Signaling (PS) and Plant RNA Regulation (PRR). Seed cassettes were prepared with dry seed and installed into the EMCS hardware on board the ISS. Experiments were initiated by remote hydration (blue arrows), and the two rotors were actuated according to the schedule shown with regular imaging (black arrows). Plant signaling **(A)** employed a constant light schedule while Plant RNA Regulation **(B)** was conducted under a 16/8 hour light/dark schedule, with two staggered hydration events allowing for the simultaneous harvest of 4 day and 6 day old seedlings at the end of the experiment.

Onboard the ISS, ECs were loaded into the EMCS by the attending astronaut. Each EC contained 5 seed cassettes for a total of 4 ECs (20 seed cassettes)/centrifuge rotor. Experiments were initiated by hydration, controlled remotely. The experimental timelines for PS and PRR are shown in **Figure 1**. Both experiments were illuminated by white LEDs along the shoot side of the cassettes, and both experiments consisted of two replicate experimental runs. PS was carried out under continuous light, while PRR employed a long-day light regime (16h light/8h dark). Images were obtained from cameras located within the EMCS chamber. EMCS environmental parameters for PS were set at 21% O_2_ and a constant seedling temperature of 24°C. For PS, the ambient cabin air was purged from the EMCS chamber prior to each experimental run so that CO_2_ levels gradually increased to equilibrate with the cabin (beginning ~370 ppm and equilibrating to ~760 ppm). EMCS environmental parameters for PRR were set at 21% O_2_, 24°C daytime seedling temperature, and 21°C nighttime seedling temperature. Due to constraints with the upmass of air gas canisters, the EMCS chamber was not purged prior to each run of PRR; chamber CO_2_ for the duration of PRR was similar to the ISS cabin (~3000 ppm). In both PS and PRR, environmental factors were monitored by real-time telemetry to record O_2_, CO_2_, seedling temperature, and relative humidity. At the end of the experiments, seed cassettes were removed from the ECs, with 1 *g* cassettes handled first, and frozen immediately in the onboard -80°C freezer (MELFI). Seed cassettes remained frozen onboard the ISS. Samples were maintained at cryogenic temperature during return via Space-X Dragon, after splashdown, and until delivery to the laboratory. Samples were then stored at -80°C until processing for RNA.

### 2.4 Ground reference controls

For both PS and PRR, ground reference experiments were carried out in the ERM-2 at NASA-ARC, using flight build spares and matching environmental parameters to the flight experiments. Briefly, this hardware was designed to replicate environmental conditions experienced aboard the ISS to include temperature, gas composition, and provide comparable support to the flight hardware. Experimental containers (ECs) were positioned in the ERM in an upright position such that seedlings grew vertically, with lighting provided within each seed cassette from above the germinating seedlings, similar to the flight configuration.

### 2.5 RNA isolation

All analyzed shoot samples for the 1 *g* controls were from seed cassettes in position 3 of each EC. For PS, position 3 corresponded to 0.76 *g* and for PRR position 3 corresponded to 1 *g* (**Supplementary Figure 1**). For each RNA isolation, seed cassettes were processed singly as follows. A single seed cassette was retrieved from the freezer, and the cover was removed. The cassette base was placed on a chilled platform, and RNA-later (Thermo Fisher Cat. No. AM7021) was added. The seedlings were dissected into root and shoot fractions and stored at 4°C in RNA-later for 24 hours, followed by storage at -20°C until RNA isolation. RNA was isolated from each shoot sample using the RNAqueous Micro kit (Applied Biosystems Cat No. AM1931). RNA recovery and integrity were monitored by Bioanalyzer (Agilent 2100). For the PRR shoot samples, the RNA isolation protocol was modified in order to retain the small RNA fraction as well.

### 2.6 Illumina sequencing

Each sample for Illumina Sequencing consisted of RNA isolated from a single seed cassette (each containing 27 seedlings) and each treatment/condition consisted of 3-4 replicates each. PS library preparation and sequencing were carried out by the Genomic Sciences Laboratory at North Carolina State University (Raleigh, NC) as described previously (Sheppard et al., 2021). Libraries were prepared using the ultra-directional library prep kit (New England Biolabs, Ipswich, MA, USA). Sequencing was carried out on 3 lanes of Illumina HiSeq2500 (125 bp single end reads). For PRR, Library preparation and Illumina sequencing for RNA and small RNA (sRNA) was carried out by Novogene Corporation Inc. (Sacramento, CA).

### 2.7 Data processing and analysis

Raw reads were cleaned up to remove adapters and reads shorter than 50 reads by cutadapt (Martin, 2011). Quality control was evaluated by FastQC (Andrews, 2010) to ensure adapters were removed. Reads were aligned using HiSat2 (Kim et al., 2019) to the Arabidopsis genome using TAIR10 (Lamesch et al., 2012) and Araport11 (Cheng et al., 2017) genome annotations (downloaded 08/2018). Read counts per feature were generated using HTseq-Count (Anders et al., 2015). Differential gene expression was determined using DESeq2 (Love et al., 2014). Features with one count or less in any sample were discarded. Differentially expressed genes were filtered on p-values. For the individual analyses of PS and PRR, the significance cutoff was padj <0.05 and for the combined analyses, the cutoff was padj < 0.01. Each sample and condition was represented by 3-4 replicates. Normalized DESeq2 counts tables were visualized as heatmaps using the R package pheatmap (version 1.0.12), clustering by column and scaling by row.

To determine whether overlap between individually analyzed PS and PRR DE gene lists were significant and non-random, hypergeometric distribution hypothesis testing was carried out using the phyper function of the R programming language. These analyses assumed a total genome size of 33,603 genes. Similarly, enrichment for HY5 targets in µ *g* DEG lists obtained from the combined PS/PRR dataset was analyzed by the same function, assuming 3,894 putative HY5 binding targets within the genome (Lee et al., 2007).

To identify potential upstream regulatory sequences present in the DEGs, a 4th order Markov background model was constructed using the 1kb regions of DNA upstream of all genes found to be differentially regulated in µ *g*. Using this model as a background control, MEME analysis (Bailey et al., 2015) examined the 500 bp regions upstream of upregulated genes using the parameters “-mod anr -minw 6 -maxw 8 -p 4 -nmotifs 10 -dna -revcomp” to elicit enriched motifs.

Gene Ontology (GO) annotation and enrichment was queried using agriGO2 (Tian et al., 2017), ExPath2.0 (Tseng et al., 2020), PANTHER18.0 (Thomas et al., 2022), and PlantGSAD (Ma et al., 2022).

PCA variance analysis was performed using variance stabilized counts from the DESeq dds object in ggplot2; this was limited to the top five hundred transcripts ranked by variance. Data was captured from the plotPCA function using ggplot_build. To determine the significance of the correlation between the variables of Age and Gravity Condition and the principal components, we used the package PCAtools R package (Blighe and Lun, 2023). The function eigencorplot was used to visualize and test the significance of the correlation between these two variables and the top six principal components (**Supplementary Figure 2**). The comparison was made using Pearson’s correlation, with pairwise.complete.obs, so that the correlation between each pair is evaluated using all paired data for each variable. The Benjamini and Hochberg multiple testing correction was applied.

### 2.8 Data availability

Raw sequence files and metadata for PS and PRR are available at the NASA data repository GeneLab under the accession numbers OSD-223 and OSD-437.

## 3 Results

### 3.1 Experimental setup and growth parameters

**Figure 2:**
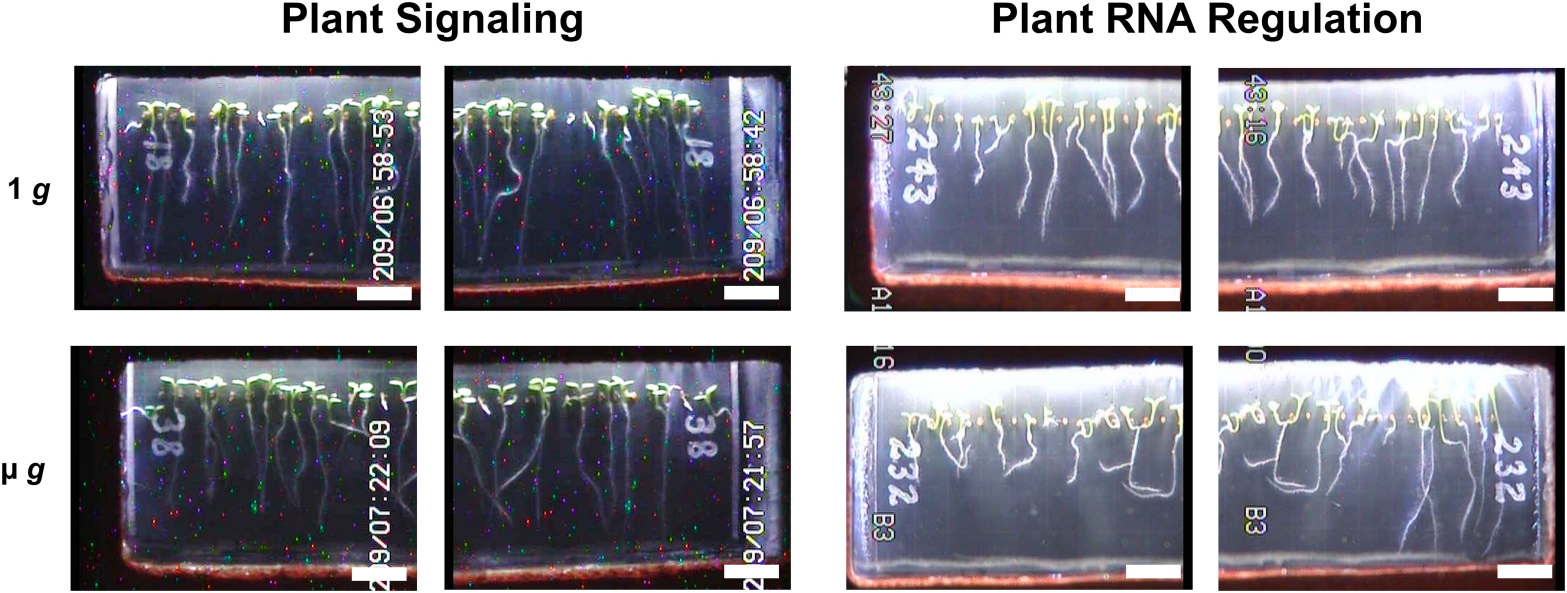
Representative Images of Plant Signaling and Plant RNA Regulation Seed Cassettes. Representative images of seedling morphology across µ *g* and 1 *g* conditions taken at the end of each experiment. Digital JPEGs are displayed directly as downlinked from EMCS hardware without alteration. Note that the camera configuration which allowed for close-up imaging provided two images of each seed cassette (left and right side), with partial overlap of each captured image (scale bar = 0.5 cm).

To minimize the impact of handling and transport, seeds were dry-mounted on membranes and remained dormant until hydration at the initiation of each experimental run. The dry seed configuration with an on-orbit hydration also eliminated the need for late loading and allowed for flexibility in the event of flight scrubbing or delayed experiment initiation. Following hydration and illumination, seedlings from PS and PRR germinated with high frequency (**Supplementary Table 1**) and exhibited nominal growth across each run of both experiments. Differences in experimental design (**Figure 1**) included the presence/absence of a day/night cycle, total experiment length, and centrifugal profiles. Notably, PS incorporated 48 hours of initial 1 *g* treatment at the start of the experiment. For the later experiment, PRR, the μ *g* rotor remained stationary for the entire duration of the experiment. A morphological difference was observed between these experiments, characterized by a largely uniform orientation of seedling roots in PS, in contrast to the more disordered growth patterns observed in PRR (**Figure 2**). This difference could be due to the initial gravitational cue provided to all seedlings in PS, the presence of a day/night cycle in PRR, or a combination of the two. Despite a small degree of variation from seed cassette to seed cassette, each experiment provided a sufficient amount of healthy plant tissue for downstream analysis, and only those cassettes which exhibited nominal growth and development were selected for downstream analysis (**Supplementary Figure 3**).

### 3.2 Differentially expressed genes

The individual DE-Seq2 analyses of PS and PRR yielded a significant number of genes that were differentially expressed between microgravity (μ *g*) and the onboard (1 *g*) control. In total, we identified 1,819 and 1,792 differentially expressed genes (DEGs) in PS, and PRR, respectively. The overlap between the two experiments for both μ *g* upregulated and μ *g* downregulated DEG lists was significant (**Table 1**). Both experiments shared 178 genes upregulated in µ *g* and 399 genes downregulated in µ *g*. GO annotation revealed enrichment of the GO terms, “Regulation of Transcription” and “Auxin Signaling” in the common µ *g* upregulated genes. “Cell Wall” and “Response to Stress” were GO terms enriched in the common µ *g* downregulated genes.

**Table 1:** Hypergeometric distribution hypothesis testing of overlap between DE genes identified by individual analysis of Plant Signaling (PS) and Plant RNA Regulation (PRR).

	PS	PRR	Overlap	Hypergeometric Test p-value
**Total DEGs (WT Shoot)**	1819	1792		
**Upregulated in micro *g***	510	599	178	9.97E-185
**Downregulated in micro *g***	1309	1193	399	1.63E-272

To further compare the two experiments, RNA-Seq reads from PS and PRR were re-analyzed in a combined study to impart increased statistical power. DE-Seq2 analysis of this combined dataset revealed 1,112 genes upregulated in µ *g* and 1,429 genes downregulated in µ *g* (**Supplementary Table 2**). It should be noted that >99% of DEGs identified as common to the two individual datasets listed above were similarly identified by this combined analysis. Additionally, this analysis further reinforced the enrichment categories initially observed by individual analyses. **Table 2** lists the major enriched GO terms for DEGs up- and downregulated in µ *g*.

**Table 2:** GO enrichment analysis of genes differentially regulated in microgravity.

Genes upregulated in microgravity					
Biological Process					
GO Accession	Description	query item	background item	p-value	FDR
**GO:0016192**	vesicle-mediated transport	52	397	1.30E-13	3.00E-10
**GO:0048193**	Golgi vesicle transport	23	108	2.80E-10	1.10E-07
**GO:0006355**	regulation of transcription, DNA-templated	155	2443	1.80E-10	7.70E-08
**GO:0048367**	shoot system development	64	896	1.70E-06	0.00019
**GO:0009734**	auxin-activated signaling pathway	23	190	2.80E-06	0.00026
**GO:0009733**	response to auxin	36	407	5.30E-06	0.00042
Cellular Compartment					
**GO:0044431**	Golgi apparatus part	83	567	8.10E-24	6.90E-21
**GO:0005794**	Golgi apparatus	123	1182	8.60E-23	3.60E-20
**GO:0012505**	endomembrane system	175	2143	1.90E-21	5.40E-19
**GO:0005802**	trans-Golgi network	45	256	5.20E-16	1.10E-13
**GO:0005634**	nucleus	487	9924	1.80E-13	1.70E-11
Genes downregulated in microgravity					
GO Accession	Description	query item	background item	p-value	FDR
Biological Process					
**GO:0006950**	response to stress	340	3506	1.60E-34	4.10E-31
**GO:0006952**	defense response	172	1566	7.80E-22	5.80E-19
**GO:0009607**	response to biotic stimulus	116	1253	1.60E-10	3.80E-08
**GO:0023052**	signaling	162	1997	3.90E-10	7.20E-08
**GO:0009414**	response to water deprivation	49	347	3.70E-10	7.10E-08
**GO:0006970**	response to osmotic stress	69	636	3.60E-09	5.20E-07
**GO:0009266**	response to temperature stimulus	63	559	4.70E-09	6.30E-07
**GO:0009737**	response to abscisic acid	62	578	3.30E-08	4.20E-06
**GO:0009651**	response to salt stress	59	574	2.60E-07	2.90E-05
**GO:0009409**	response to cold	45	384	2.70E-07	2.90E-05
**GO:0071554**	cell wall organization or biogenesis	61	704	2.50E-05	0.0018
Cellular Compartment					
**GO:0009536**	plastid	293	4213	1.80E-10	2.10E-08
**GO:0009507**	chloroplast	289	4148	2.10E-10	2.10E-08
**GO:0009579**	thylakoid	66	584	1.80E-09	1.50E-07
**GO:0005618**	cell wall	68	706	3.00E-07	1.10E-05

#### 3.2.1 Genes upregulated in μ *g*, compared to 1 *g* in space

##### 3.2.1.1 Transcription factors

Similar to the individual studies, the combined results for PS and PRR show strong enrichment of genes associated with DNA transcription. In total, 159 Transcription Factors (TFs) as identified by Pruneda-Paz et al. (Pruneda-Paz et al., 2014) were upregulated in µ *g* (**Supplementary Table 3**). The majority of these TFs are involved in regulating shoot meristem and leaf growth and are associated with organ patterning, leaf development, and photomorphogenesis. Specifically, *SPCH*, *WOX3*, *TCP22*, *GATA2*, *HFR1*, several *GRF*s, as well as TFs mediating auxin responses and brassinosteroid (BR) signaling were upregulated in the μ *g* condition relative to 1 *g* controls. The upregulated list also included 8 TFs belonging to the Apetala2/Ethylene response factor (AP2/ERF) superfamily, of which cytokinin response factors (CRF4 and CRF5) and Aintegumenta (ANT) play established roles in cell proliferation and embryo, cotyledon and leaf development (Mizukami and Fischer, 2000; Rashotte et al., 2006).

##### 3.2.1.2 Other transcriptional regulators

Among the DEGs upregulated in μ *g*, 7 genes with potential epigenetic regulatory function were noted. Of these, 5 genes encoded SET domain containing histone methyltransferases including 3 members of the SUVH family (Naumann et al., 2005). Genome wide methylation changes leading to altered transcriptional profiles have been reported in response to spaceflight (Zhou et al., 2019; Paul et al., 2021).

##### 3.2.1.3 Auxin related genes

Another conserved category that was prominent in the combined analysis is auxin transport and signaling (**Table 3**); this group includes 2 auxin efflux carriers (*PIN3* and *PIN4*), six auxin response factors (*ARF*s), and 11 small auxin upregulated (*SAUR*) genes.

**Table 3:** Auxin related genes up regulated in microgravity.

Gene ID	Log2 Fold Change	padj	Symbol	Description
**AT1G70940**	0.4567	1.46E-03	*PIN3*	Auxin efflux regulator
**AT3G26810**	0.2620	7.66E-03	*AFB2*	Auxin F-box protein
**AT2G33310**	0.4547	3.04E-04	*IAA13*	Auxin induced protein
**AT1G12820**	0.3672	3.25E-03	*AFB3*	Auxin signaling F-box protein
**AT1G31880**	0.7857	5.47E-03	*BRX*	BRX family protein
**AT5G47750**	0.2417	2.70E-03	*D6PKL2*	D6PK family protein kinase
**AT1G16510**	1.5668	6.93E-08	*SAUR41*	Clade III SAUR gene
**AT3G62150**	0.6457	2.42E-03	*ABCB21*	ATP-binding cassette transporter
**AT5G60450**	0.4424	9.39E-03	*ARF4*	Auxin response factor
**AT1G59750**	0.3375	8.86E-05	*ARF1*	Auxin response factor
**AT1G30330**	0.8869	6.11E-06	*ARF6*	Auxin response factor
**AT1G15750**	0.2680	6.81E-05	*TPL*	WUS-interacting protein
**AT2G01420**	0.5521	1.34E-04	*PIN4*	Auxin efflux carrier
**AT5G59430**	0.4832	2.88E-03	*TRP1*	Telomere repeat binding protein
**AT1G77850**	0.5913	9.30E-07	*ARF17*	Auxin response factor
**AT3G62980**	0.3926	2.95E-04	*TIR1*	Auxin receptor
**AT1G19220**	0.4469	9.25E-03	*ARF19*	Auxin response factor
**AT3G59900**	0.7563	9.71E-04	*ARGOS*	Auxin-Regulated Gene Involved in Organ Size
**AT5G47370**	1.8362	5.23E-13	*HAT2*	Homeobox-leucine zipper
**AT4G32280**	2.6829	2.07E-08	*IAA29*	Auxin induced protein
**AT4G37580**	1.2138	1.91E-04	*HLS1*	Putative N-acetyltransferase
**AT4G30080**	0.6009	2.40E-04	*ARF16*	Auxin response factor
**AT4G38850**	1.6286	2.91E-04	*SAUR15*	SAUR-like auxin-responsive protein
**AT3G16500**	1.0398	2.51E-10	*PAP1*	Phytochrome-associated protein
**AT5G24520**	0.3512	1.89E-03	*TTG1*	WD40 repeat containing protein
**AT5G12050**	1.0703	4.08E-06	*BG1*	Rho GTPase-activating protein
**AT2G45210**	1.2634	1.12E-04	*SAUR36*	SAUR-like auxin-responsive protein
**AT4G34770**	0.9559	5.70E-03	*SAUR1*	SAUR-like auxin-responsive protein
**AT4G38825**	2.1098	3.04E-05	*SAUR13*	SAUR-like auxin-responsive protein
**AT4G38840**	0.8468	3.04E-05	*SAUR14*	SAUR-like auxin-responsive protein
**AT4G38860**	1.3374	3.72E-09	*SAUR16*	SAUR-like auxin-responsive protein
**AT1G75580**	1.5290	7.84E-06	*SAUR51*	SAUR-like auxin-responsive protein
**AT3G60690**	1.0297	3.73E-03	*SAUR59*	SAUR-like auxin-responsive protein
**AT5G20820**	3.1398	9.25E-04	*SAUR76*	SAUR-like auxin-responsive protein
**AT4G36110**	1.7236	2.51E-04	*SAUR9*	SAUR-like auxin-responsive protein
**AT1G69160**	0.8261	4.53E-03	*BGL1*	BIG GRAIN like protein
**AT2G42620**	0.6706	2.67E-05	*MAX2*	F-box leucine-rich repeat family protein
**AT5G08560**	0.2504	5.05E-03	*WDR26*	WD40 repeat containing protein

*SAUR*s were among the more highly upregulated genes in µ *g*, with 9 of the 11 induced more than 2-fold. *SAUR*s were first characterized as rapidly induced in response to auxin, although a few *SAUR*s are repressed by auxin (Ren and Gray, 2015). Of the 11 *SAUR*s identified in this dataset, 10 are induced by auxin; *SAUR15* and *SAUR16* may also be induced by Gibberellic acid (GA) and Brassinosteroids (Ren and Gray, 2015). SAURs 13-16 are classified as lirSAURs (Sun et al., 2016) that are light-regulated in cotyledons and hypocotyls and directly interact with phytochrome interacting factors (PIF)s (Dong et al., 2019). Overexpression of *SAUR36* and *SAUR41* increases cell expansion and promotes hypocotyl elongation (Chae et al., 2012; Stamm and Kumar, 2013). SAUR36 is implicated in leaf growth senescence (Hou et al., 2013), and SAUR76 may act as a negative regulator of leaf expansion by regulating cell division (Markakis et al., 2013). SAUR15 is involved in the shade avoidance response and is a potential target of PIF4. Interestingly, *SAUR15* expression has been shown to increase on the lower flank of inflorescence stems 30 min following gravistimulation (Taniguchi et al., 2014).

We also detected 6 *ARF*s upregulated in µ *g*. In general, ARFs are TFs that are bound by Aux/IAA repressors and remain inactive in the absence of auxin. This repression is relieved in the presence of auxin, promoting transcriptional activation of auxin-responsive downstream genes. Three of the *ARF*s detected in this dataset (*ARF1*, *ARF4* and *ARF19*) are implicated in leaf development, while ARF6 and ARF17 play a role in flowering (Cancé et al., 2022; Li et al., 2023).

##### 3.2.1.4 Vesicle transport genes

Interestingly, the combined analysis of genes enriched in µ *g* identified a third enriched group –genes involved vesicle-mediated transport– that was not detected in the individual analyses of each experiment. This category consists of approximately 50 genes, including those encoding for members of the coat protein (COP) complex, Sec23/24 protein transport, and several proteins associated with Golgi and the endomembrane system (Hwang and Robinson, 2009). The enrichment of vesicle transport is consistent with observation that shoot growth and developmental processes appear to be altered under µ *g*.

Not surprisingly, given the enrichment of biological processes described above involving vesicle transport, auxin responses, and transcriptional regulators, the cellular compartments overrepresented in the µ *g* upregulated dataset are Golgi and the nucleus (**Table 2**).

##### 3.2.1.5 Promoter analysis of genes upregulated in µ *g*

**Figure 3:**
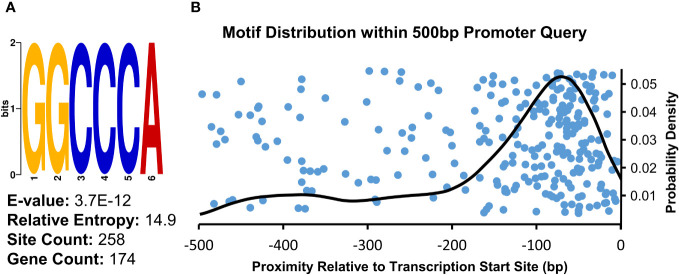
Consensus Sequence and Distribution of the Site II Element Enriched in Promoter Regions of Upregulated Transcripts. **(A)** Sequence analysis of the 500 bp region upstream of µ *g* upregulated transcripts showed an enrichment for the motif identified as the Site II element. **(B)** Density analysis of the distribution of this motif within the analyzed region indicates a probability density maxima at -70bp relative to transcriptional start sites (right). The position of individual occurrences of this motif within the analyzed regions is plotted along the x-axis (blue dots), visualized with an artificial spread across the y-axis.

To examine whether any *cis*-regulatory elements may be associated with the genes upregulated in the µ *g* condition relative to onboard 1 *g* controls, we analyzed regions upstream of the transcript initiation site using Multiple Expectation maximizations for Motif Elicitation (MEME) software. A consensus sequence, 5’-GGCCCA-3’, was returned as the top result of this query, with an E-value of 3.7E-12 (**Figure 3A**). There were 258 occurrences of this motif in 174 unique genes (15.6% of total input genes). Further, this motif predominantly occurred in the -100 to -0 bp region upstream of transcript initiation sites, as seen in the density analysis (**Figure 3B**). This motif, also known as ‘Up1’’(GGCCCAWWW) or ‘‘Site II element’’ (TGGGCY), is overrepresented in the -200 to -1 bp region of Arabidopsis transcripts (Molina and Grotewold, 2005; Davis et al., 2012). Additionally, this sequence has been identified as a binding site of the TB1, CYCLOIDEA, PCF (TCP) plant-specific family of transcription factors (Viola et al., 2011). Arabidopsis TCP proteins regulate many aspects of development including cell cycle control, leaf morphology, senescence as well as defense responses (Aguilar Martinez and Sinha, 2013; Li, 2015). Consistent with the enrichment of this element upstream of genes upregulated in µ *g*, a class I TCP (*TCP22*) was found to be upregulated in µ *g* (**Supplementary Table 3**).

#### 3.2.2 Genes downregulated in µ *g*, compared to 1 *g* in space

The combined analysis of PS and PRR indicated that responses to stress (both abiotic and biotic) were highly enriched in genes downregulated in µ *g* (**Table 2**), consistent with what was observed in each individual experiment. This overall dampening of stress response genes includes a downregulation of genes encoding several members of Cytochrome P450 family, 14 Glutathione S-transferases, and members of a MATE efflux family involved in detoxification. In addition, we detected an enrichment of plastid-associated genes as well as several involved in cell wall organization.

##### 3.2.2.1 Transcription factors

**Figure 4:**
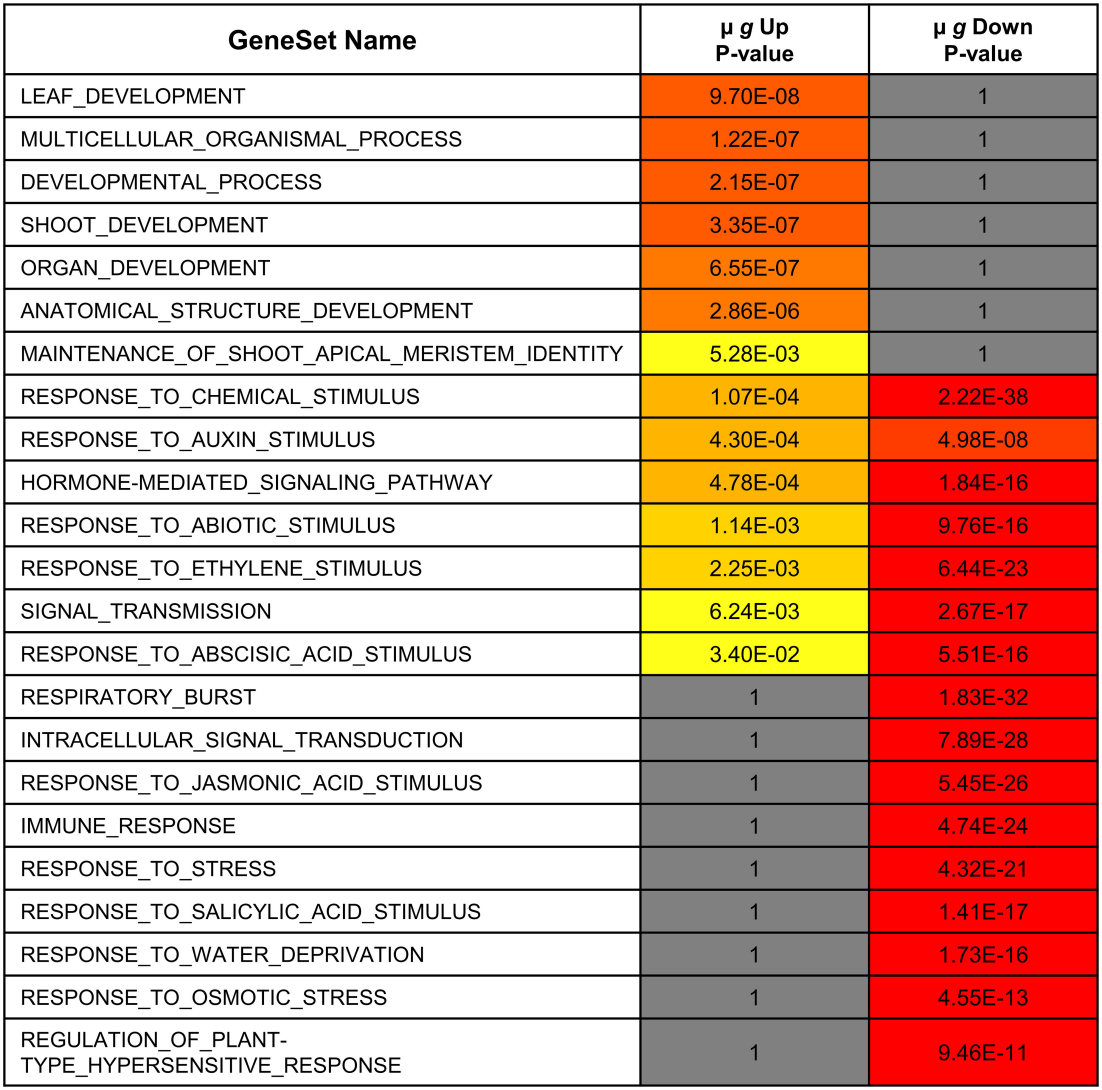
Functional comparison of transcription factors differentially expressed in microgravity. Differentially regulated transcription factor gene lists were analyzed for enrichment by PlantGSAD. Following GO enrichment, Singular Enrichment Analyses (SEA)s were compared by SEACOMPARE to identify common and/or unique GO categories using the online tool in PlantGSAD. The p values for GO categories were generated by the SEACOMPARE tool and represent the significance of the GO terms. The color progression from yellow to red represents increasing significance while the gray bars are not significant.

The µ *g* downregulated DEGs include 120 TFs (**Supplementary Table 4**). Unlike the upregulated TFs, which were associated with developmental responses, the downregulated TFs are primarily involved in regulating stress responses (**Figure 4**). The downregulated TFs include those implicated in biotic stress and plant defense (Ng et al., 2018) and those involved in abiotic stresses, including osmotic and temperature stress. As is illustrated in **Figure 4**, the functions of these downregulated TFs are distinct with limited overlap with those of the upregulated TFs. While 4-5 GO terms show some overlap, these categories are most significant in the downregulated TFs. Further analysis within the overlapping categories show for instance that the auxin and ABA related terms in the upregulated TF group are involved with growth while the downregulated TF genes in these groups contain those that regulate stress as described below.

The largest groups of TF downregulated in µ *g* are 15 ethylene response factors (ERF)s and 12 WRKY TFs (Phukan et al., 2016). The WRKY TFs in our dataset appear to function chiefly in abiotic or biotic stress responses. The ERFs include 7 dehydration responsive element-binding (DREB) subfamily A members including CBF2, (Shinozaki and Yamaguchi-Shinozaki, 2000; Novillo et al., 2004) and 8 AP2/ERF family TFs (Srivastava and Kumar, 2019). The downregulated TF list also includes 4 heat stress TFs, several MYB and NAC family TFs, and calmodulin binding transcriptional activators (CAMTA)s.

##### 3.2.2.2 Biotic stress and defense related genes

Among the genes downregulated in µ *g*, approximately 200 genes were associated with plant defense and response to biotic stimuli. This category includes 24 disease-resistance proteins, different classes of receptor-like protein kinases, and several WRKY TFs. Of interest, it has been reported that WRKY18, WRKY40 and WRKY60 (which are represented in our dataset) form a cluster that work either synergistically or antagonistically in response to bacterial or necrotrophic pathogens (Xu et al., 2006; Schön et al., 2013).

##### 3.2.2.3 Abiotic stress related genes

In addition to the downregulation of genes involved in biotic stress responses, we also detected approximately 140 downregulated genes associated with the responses to temperature stimulus and ABA-mediated regulation of osmotic stress (**Supplementary Table 5**). Song et al. carried out a time series of RNA-Seq experiments and large-scale ChIP-Seq focused on 21 ABA-responsive TFs (Song et al., 2016). This study revealed a TF hierarchical network regulating ABA responses in Arabidopsis. Interestingly, 9 of these 21 TFs (namely, *NFY-B2*, *NFY-C2*, *GBF3*, *MYB44*, *DIV2*, *HB7*, *NAC032*, *NAC102* and *RD26*) are downregulated in our dataset.

##### 3.2.2.4 Calcium signaling related genes

Given the enhancement in stress responses, it is not surprising that approximately 40 genes involved in calcium signaling were downregulated in µ *g* (**Table 4**). This category includes calcium binding proteins, calcium-dependent protein kinases (CDPK)s, calmodulin-binding proteins and TFs, CBL-interacting protein kinases, and cyclic nucleotide gated ion channels (CNGC)s.

**Table 4:** Ca^++^ related genes downregulated in microgravity.

Gene ID	Log2 Fold Change	padj	Symbol	Description
**AT4G17615**	-0.5692	1.08E-03	*CBL1*	Calcineurin B-like protein 1
**AT5G66650**	-1.4308	3.72E-04	*CMCU*	Calcium uniporter protein 3
**AT4G32060**	-0.5073	3.60E-04	*MICU*	Calcium uptake protein, mitochondrial
**AT5G54590**	-0.6115	5.94E-03	*CRLK1*	Calcium/calmodulin-regulated receptor-like kinase
**AT5G37770**	-1.7365	4.38E-09	*CML24*	Calcium-binding protein
**AT5G42380**	-2.7770	8.69E-03	*CML37*	Calcium-binding protein
**AT5G49480**	-1.0429	5.98E-06	*CP1*	Calcium-binding protein
**AT2G46600**	-2.6536	8.42E-14	*KIC*	Calcium-binding protein
**AT4G27280**	-3.6521	2.59E-10	*KRP1*	Calcium-binding protein
**AT5G54490**	-2.2801	3.69E-06	*PBP1*	Calcium-binding protein
**AT3G14590**	-1.1538	2.31E-04	*NTMC2T6.2*	Calcium-dependent lipid-binding family protein
**AT4G21940**	-0.8663	2.51E-03	*CPK15*	Calcium-dependent protein kinase
**AT5G66210**	-1.7608	1.82E-09	*CPK28*	Calcium-dependent protein kinase
**AT1G76040**	-0.9149	3.36E-03	*CPK29*	Calcium-dependent protein kinase
**AT3G57530**	-1.9357	1.37E-11	*CPK32*	Calcium-dependent protein kinase
**AT4G09570**	-0.4804	3.47E-03	*CPK4*	Calcium-dependent protein kinase
**AT1G27770**	-1.8644	2.82E-11	*ACA1*	Calcium-transporting ATPase
**AT2G41010**	-1.5839	2.42E-12	*CAMBP25*	Calmodulin-binding protein
**AT5G62570**	-1.4615	3.55E-08	*CBP60A*	Calmodulin-binding protein
**AT2G24300**	-1.0421	3.92E-03	*CBP60E*	Calmodulin-binding protein
**AT5G26920**	-2.8406	1.07E-06	*CBP60G*	Calmodulin-binding protein
**AT5G64220**	-0.4456	1.03E-04	*CAMTA2*	Calmodulin-binding transcription activator
**AT2G22300**	-0.5213	1.30E-03	*CAMTA3*	Calmodulin-binding transcription activator
**AT1G67310**	-0.4575	3.13E-05	*CAMTA4*	Calmodulin-binding transcription activator
**AT2G41100**	-1.4492	5.29E-06	*CML12*	Calmodulin-like protein
**AT2G43290**	-1.7077	5.75E-14	*CML5*	Calmodulin-like protein
**AT3G51920**	-0.8321	5.20E-06	*CML9*	Calmodulin-like protein
**AT1G01130**	-0.9225	5.87E-05	*CIPK*	CBL-interacting serine/threonine-protein kinase
**AT2G30360**	-0.7795	9.29E-05	*CIPK11*	CBL-interacting serine/threonine-protein kinase
**AT5G01820**	-0.7076	1.98E-03	*CIPK14*	CBL-interacting serine/threonine-protein kinase
**AT1G48260**	-0.7532	6.49E-03	*CIPK17*	CBL-interacting serine/threonine-protein kinase
**AT5G07070**	-0.7082	5.74E-05	*CIPK2*	CBL-interacting serine/threonine-protein kinase
**AT3G19100**	-0.4980	3.52E-04	*CRK2*	CDPK-related kinase
**AT5G53130**	-1.2025	1.84E-07	*CNGC1*	Cyclic nucleotide-gated ion channel
**AT5G54250**	-0.8611	5.00E-05	*CNGC4*	Cyclic nucleotide-gated ion channel

#### 3.2.3 Non-coding RNAs

Natural antisense transcripts (NAT)s are antisense sequences which may overlap in part with protein coding (sense) transcripts. This sense-antisense pairing can lead to regulation of the sense transcript (Wight and Werner, 2013). A negative correlation in expression (i.e. NAT up and sense transcript down) may be indicative of gene silencing; however, NATs may also enhance sense transcript expression by regulating mRNA stability and/or translation (Reis and Poirier, 2021).

Among the DEGs in µ *g*, we found 42 upregulated NATs and 30 downregulated NATs. Interestingly, we observe a negative correlation between NAT expression and the overlapping sense transcripts for 29 of the 42 upregulated NATs suggesting a possible means of repression of these genes. Among these are several genes encoding for chloroplastic proteins, defense related proteins and the TF *MYB44* (**Supplementary Table 6**). Additionally, we observed that the expression of 20 of the overlapping sense transcripts for the 30 downregulated NATs are upregulated under µ *g* (**Supplementary Table 6**).

### 3.3 Microgravity versus the space environment

**Figure 5:**
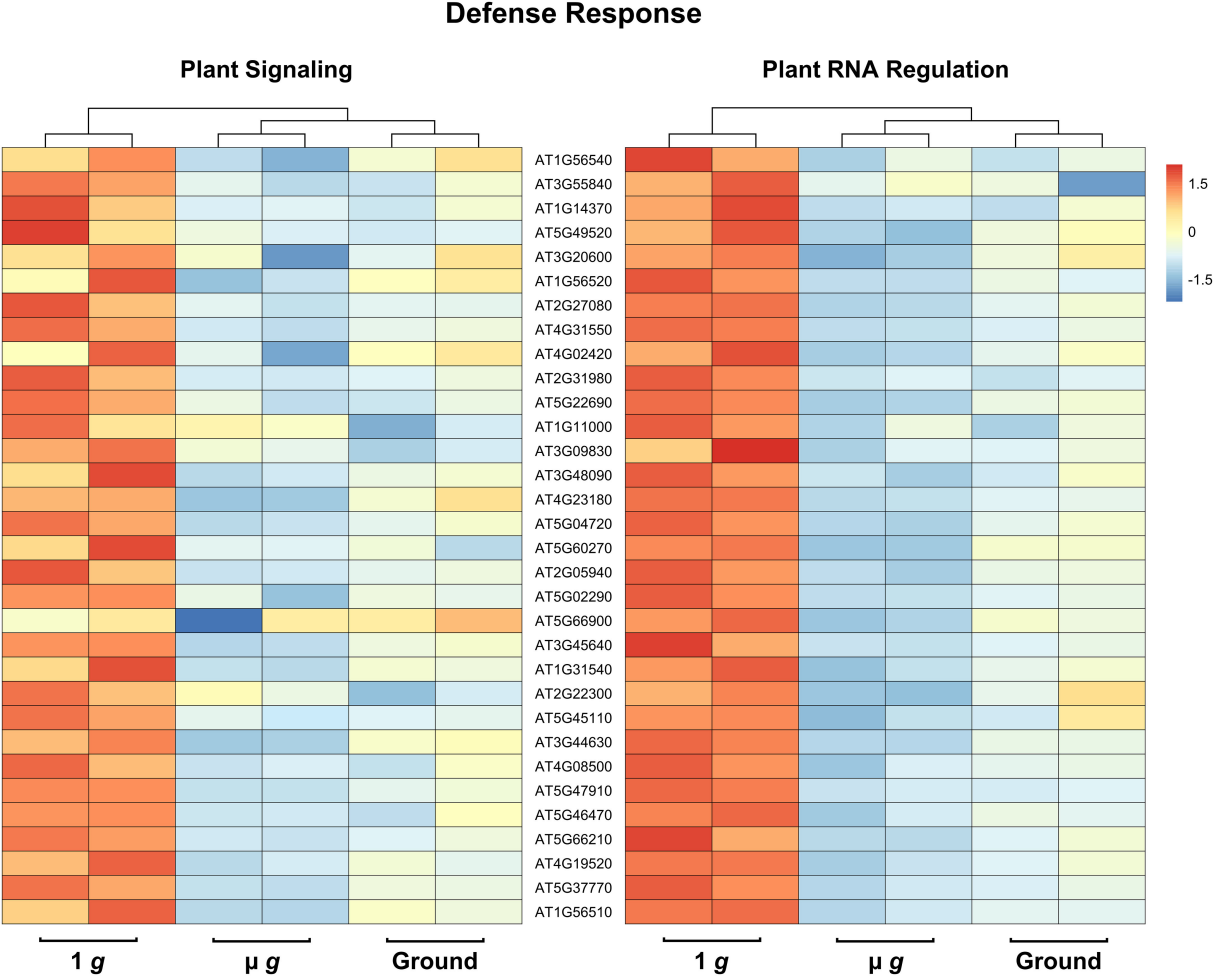
Expression profiles of DEG subsets across 1 *g*, µ *g*, and ground control conditions. DESeq2 normalized counts (two replicates for each condition) of defense DEG subsets were visualized as heatmaps by the R package pheatmap. Data presented are clustered by column (dendrogram, top), and scaled by row.

**Figure 6:**
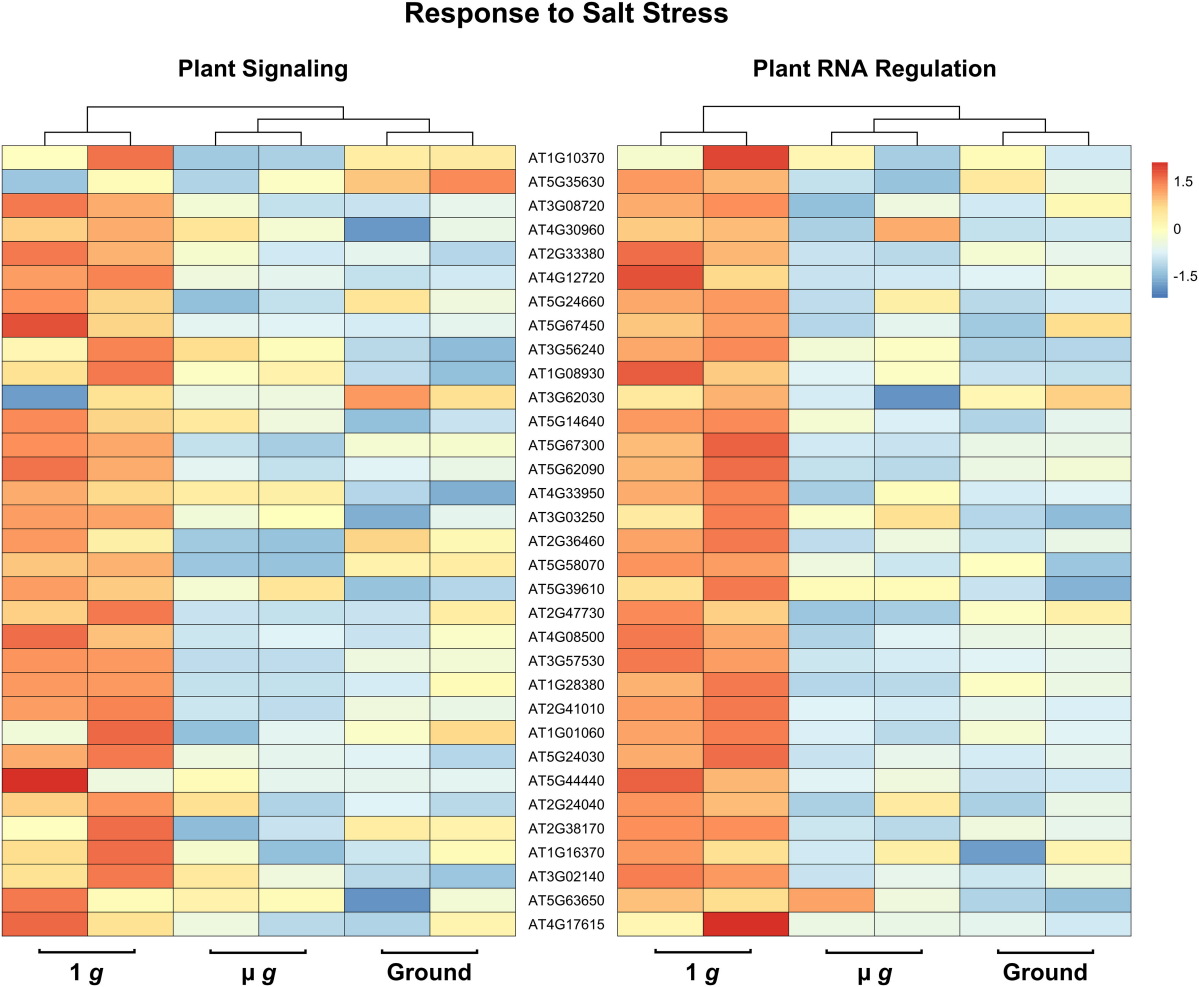
Expression profiles of DEG subsets across 1 *g*, µ *g*, and ground control conditions. DESeq2 normalized counts (two replicates for each condition) of salt stress DEG subsets were visualized as heatmaps by the R package pheatmap. Data presented are clustered by column (dendrogram, top), and scaled by row.

Perhaps the most striking aspect of the DEGs that are downregulated in both PS and PRR is that > 400 genes are associated with stress responses. This included a strong enrichment of biotic stress response (in particular, response to bacteria and chitin) and genes known to respond to many abiotic stresses, including salt and temperature. Curiously, these responses have been reported as upregulated in spaceflight experiments compared to ground controls. It is not surprising that the space environment poses several challenges to plants and many of these are not easy to measure or replicate on the ground. Although our focus in both PS and PRR spaceflights was the direct comparison of µ *g* with the onboard 1 *g* control, we also carried out ground controls where EMCS chamber conditions were matched in the ground reference module ERM-2. An examination of the DEGs between 1 *g* in space and ground controls revealed that many of these stress response-related genes were actually upregulated in 1 *g* compared to the ground. These observations indicate that the space environment (excluding µ *g*) enhanced the expression of these stress-related genes. However, this group of genes was specifically downregulated in µ *g* compared to 1 *g* suggesting that microgravity masks the induction of these genes in the space environment. Representative heat maps for select defense-related genes are shown in **Figure 5** (**Supplementary Table 7**). As an example of an abiotic stress response, heat maps for a set of genes involved in salt stress are shown in **Figure 6** (**Supplementary Table 8**). These heat maps clearly illustrate that the stress-related genes showed higher levels of expression in the spaceflight 1 *g* condition compared to either µ *g* or the ground controls. Since the 1 *g* and µ *g* experiments were conducted on two rotors held in the same chamber at the same time, we can be confident that all other “space-related” stimuli were shared between the two, and that the primary difference between them was the µ *g* treatment. The differences between 1 *g* and the ground control samples, would include all other effects of the space environment, excluding microgravity. It is clear that many of these genes would not have been detected as differentially expressed in the absence of the on board 1 *g* control and these results reveal that this control is critical in order to identify µ *g* specific changes.

#### 3.3.1 Micro RNAs detected and their target genes

In addition to mRNA profiling, the PRR spaceflight experiment included sequencing of small RNAs (sRNA)s which revealed that several micro RNAs (miRNA)s were differentially regulated in µ *g* compared to 1 *g* and ground controls. miRNAs act to regulate gene expression either by the cleavage of their target mRNAs, or by repression of translation or DNA methylation (Song et al., 2019).

**Figure 7:**
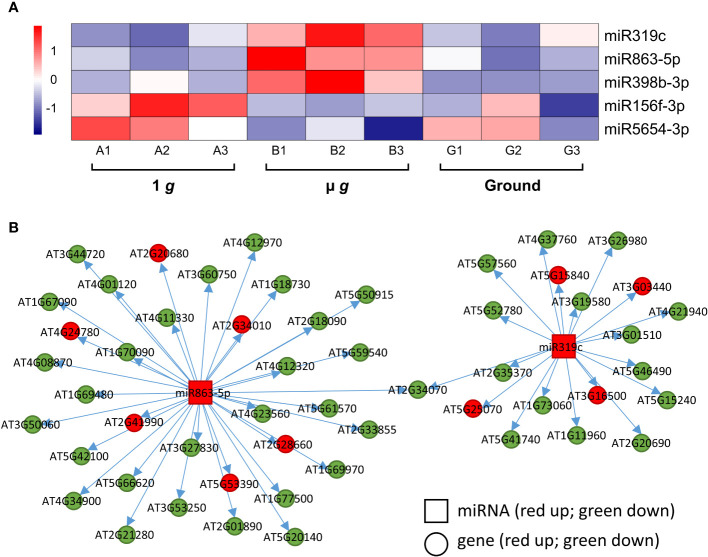
Differentially expressed miRNAs and their target genes. **(A)** Heatmap showing the expression of differentially regulated miRNAs. **(B)** Correlation of expression between miR863-5p and miR319c and their putative targets. Squares denote the miRNAs and circles represent the miRNA target genes. Red and green represent up and down regulation, respectively.

We identified 3 miRNAs –miR319c, miR863-5p and miR398b-3p– that were upregulated in µ *g* (**Figure 7A**). Both miR863 and miR398b are found in Arabidopsis leaves and seedlings (Meng et al., 2012). The miR398 family was shown to target Cu^++^ and Zn^++^ dismutase enzymes in response to Cu^++^ deficiency (Yamasaki et al., 2007) and the miR319 family is involved in the regulation of leaf shape via their interaction with TCP TFs (Bresso et al., 2018). Although the specific targets listed in these publications were not represented in our dataset, we did detect several putative miRNA targets. Correlation analysis of miR319c and miR863-5p and their predicted mRNA targets detected in our dataset are shown in **Figure 7B** (**Supplementary Table 9**). It can be seen that many of the predicted targets were coordinately downregulated in µ *g*, consistent with repression by these miRNAs. The miR319c cluster of downregulated targets include several defense-related genes and 12 of the 22 miR398b-3p target genes encode for chloroplastic enzymes and proteins. Interestingly, overall 38% of the downregulated miR319c, miR863-5p and miR398b-3p target gene products are localized in the plastid.

Two miRNAs, miR156f-3p and miR5654-3p were upregulated in 1 *g* relative to µ *g* (**Figure 7A**) with coordinate down regulation of 14-16 of their predicted targets in 1 *g* (**Supplementary Table 9**). The miR156 family is closely associated with and regulates several Squamosa Promoter binding Like (SPL) TFs and is important for regulating phase transitions such as the progression from juvenile to adult stages and flowering time (Wang and Wang, 2015). Most recently, miR156 has been shown to modulate seedling growth in response to temperature and light changes via its interaction with SPL9 (Sang et al., 2023). Coincidentally, *SPL9* expression is downregulated in 1 *g* in our dataset along with other targets of miR156f-3p, which are related to auxin and ethylene signaling.

We also observed that both µ *g* and 1 *g* seedlings had higher expression of miR398b-3p compared to ground control and 4 miR398b-3p target genes were coordinately downregulated in both µ *g* and 1 *g* compared with ground, including two TFs, *GTE11* and *ATRX-like-protein*.

#### 3.3.2 Day 4 versus day 6: a possible interaction between µ *g* responses and development

**Figure 8:**
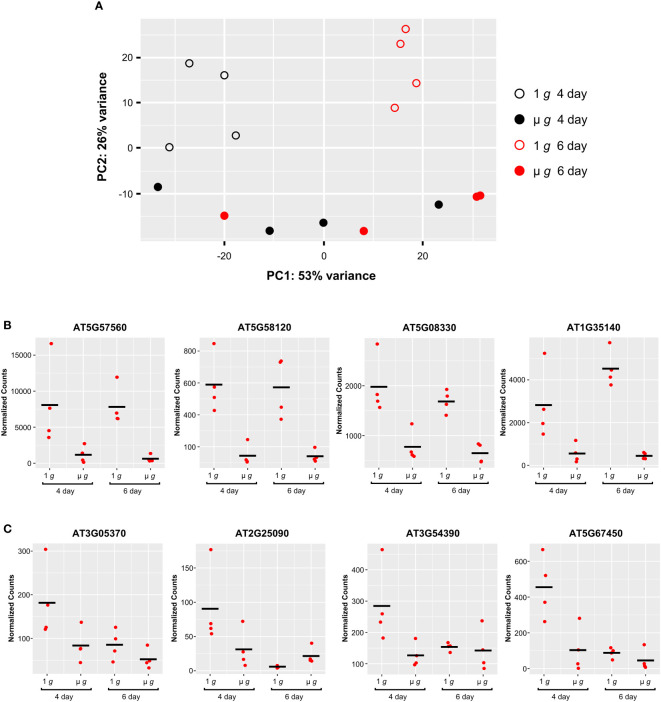
Comparison of Expression Profiles at Day 4 and Day 6. **(A)** Principal component analysis (PCA) of the PRR flight samples. Filled and open symbols represent µ *g* and 1 *g*, respectively (black circles, Day 4; red circles, Day 6). **(B)** Representative examples of individual genes that show similar patterns of expression between 1 *g* and µ *g*, at Day 4 and Day 6. **(C)** Representative examples of individual genes showing higher expression uniquely at Day 4 in 1 *g*. DESeq2 normalized counts were visualized using ggplot2.

The experimental design of PRR with staggered hydration allowed for some samples to be initiated 48h later, yielding two time points (Day 4 and Day 6) from the same experiment. Our major focus was on DEGs detected at Day 6, described in the preceding sections. Comparing the effects of microgravity on the Day 4 and Day 6 plants revealed interactions between plant developmental stage and microgravity. These interactions are apparent in the PCA plot of the PRR Day 4 and 6 flight samples (**Figure 8A**). Principal component 1 primarily separated Day 4 and Day 6 samples under 1 *g* (p<0.05). Principal component 2 separated 1 *g* and µ *g* at either day (p<0.001). However, there was little separation between the Day 4 and Day 6 samples in µ *g* in the first principal component (**Figure 8A**).

Examination of the DEGs at Day 4 revealed that a much smaller fraction of genes were differentially regulated in µ *g* at Day 4 (compared with the DEGs detected at Day 6). In total, at Day 4 there were 406 DEGs (75 up and 331 down in µ *g* compared to 1 *g*). Many of these DEGs (~ 40% of the up- and 60% of downregulated genes in µ *g*) at Day 4 were shared with the Day 6 data. A majority of the 210 genes commonly downregulated in µ *g* at both Day 4 and Day 6, were related to abiotic and biotic stresses as described previously. This finding indicates that µ *g* specific downregulation of stress responses occurs as early as Day 4 and is common between the two times sampled. Genes associated with cell wall organization were also found to be downregulated at both days. Examples of genes which exhibit similar downregulation in microgravity at both Day 4 and 6 are depicted in **Figure 8B**.

We also observed that several genes were uniquely downregulated in µ *g* at Day 4 only. Scatter plots of representative genes in this group clearly indicate that the differential expression observed at Day 4 did not persist at Day 6 (**Figure 8C**).

Taken together, these results suggest that the developmental window the samples are in could influence the observed transcription response profiles and point to interactions between µ *g* and the developmental stage.

## 4 Discussion

### 4.1 Conserved and unique transcriptional changes in microgravity

The PS and PRR spaceflight experiments were designed to examine early seedling development and responses to microgravity. To answer these questions, we utilized the EMCS facility with a rotor configuration selected to provide µ *g* and an onboard 1 *g* control. The two experiments were conducted at different times and had different lighting regimes as well as some other differences in set up as outlined in **Figure 1**. Despite these differences, there was considerable parity between the DEGs in µ *g* between the two experiments. Genes upregulated in µ *g* compared to the 1 *g* control were primarily involved in leaf and shoot development. The majority of the upregulated TFs function to regulate these processes and the enrichment in auxin and vesicle transport also support growth processes. In contrast, there appears to be a substantial downregulation of both biotic and abiotic stress responses in µ *g*. Defense related genes were overrepresented and most of the downregulated TFs function in mediating plant stress responses (**Figure 4**). Additionally, we identified specific miRNAs that were differentially regulated in microgravity with coordinate regulation of their putative targets.

The large-scale downregulation in microgravity of stress-response related genes in the PS and PRR flight experiments are somewhat at odds with other experiments where flight responses are compared to a ground control on Earth. In fact, we find that genes reported as upregulated in spaceflight appear to be downregulated in our datasets. For instance, 70 genes that were upregulated in spaceflight in BRIC19 (Choi et al., 2019) are downregulated in µ *g* in our dataset. Many of these common genes are associated with response to stress and hypoxia.

We do however find more overlap in responses if we examine RNA-Seq datasets from other EMCS spaceflight experiments which utilized onboard 1 *g* controls. The Seedling Growth experiments (SG2 and SG3) were conducted in the EMCS; however these experiments included photostimulation with either unidirectional blue (Herranz et al., 2019; Vandenbrink et al., 2019) or red (Villacampa et al., 2021) light for the final 48h of the experimental timeline. Although there was almost no overlap in DEGs upregulated in µ *g*; we do detect considerable overlap with µ *g* downregulated genes as well as genes downregulated in low (<0.1) *g* (Herranz et al., 2019). Comparing our results to Herranz et al. shows that 54 genes were commonly downregulated in µ *g*. Furthermore, 143 genes downregulated in low *g* were shared with genes downregulated in µ *g* in our data. Even more pronounced is the similarity in enriched GO terms for genes downregulated in µ *g* and low *g* compared to 1 *g* (Herranz et al., 2019) and the genes downregulated in µ *g* in the PS/PRR data. These include, response to stimulus, response to abiotic stimulus and response to stress. Genes downregulated in µ *g* in with the red light photostimulation (Villacampa et al., 2021) were enriched in the GO categories response to bacterium and fungus. Taken together these results further reinforce the idea that both biotic and abiotic stress responses appear to be specifically downregulated in µ *g*. This regulation would not have been detected without the onboard 1 *g* condition for comparison and these results point to the value of having an onboard 1 *g* control.

At least 4 different Arabidopsis ecotypes have been flown in space including Col-0, Ler, Ws and Cvi (Paul et al., 2017; Choi et al., 2019). Interestingly, the Col-0 ecotype was found to be more responsive to the spaceflight environment compared to both Ws and Ler with more DEGs detected overall. A direct comparison of our results (on light grown Col-0 shoots) with the previous studies is hampered by the fact that they utilized either root tips or etiolated whole seedlings. Nevertheless, when we compare our findings with other spaceflight experiments, we find that two common features stand out; many experiments have reported altered expression of genes involved in cell wall modification and photosynthesis/plastid related genes. Two unexpected findings of our experiments are the potential regulation by the transcription factor Elongated Hypocotyl 5(HY5) and the altered expression of a few gravitropism related genes.

#### 4.1.1 Cell wall related genes

Genes involved in remodeling of plant cell walls have been reported to be differentially expressed in previous spaceflight experiments (Paul et al., 2013; Kwon et al., 2015; Johnson et al., 2017; Choi et al., 2019; Nakashima et al., 2023). Glycomic analysis of spaceflight plant samples have shown changes in cell wall glycans, consistent with spaceflight related modification of cell walls (Johnson et al., 2017; Nakashima et al., 2023). Similar to these previous reports, we also observed that genes involved in cell wall modification are overrepresented in our datasets; particularly we found approximately 100 genes associated with cell wall organization and modification downregulated in µ *g*. These included genes encoding for several members of the xyloglucan endotransglucosylase (XTH) family, expansins, peroxidases, pectin esterases and polygalacturonases. These cell wall proteins are involved in cell wall polymer rearrangement which occurs during cell elongation (Irshad et al., 2008). The individual genes in these enzyme categories however, were not conserved between our dataset and the previous reports; probably due to differences in tissue type, age and growth conditions. We also found cellulose synthase-like genes and 6 genes encoding for dirigent proteins which are involved in lignin biosynthesis to be downregulated in µ *g*.

#### 4.1.2 Chloroplast and photosynthesis related genes

The mis-regulation or altered regulation of photosynthesis related genes has been reported for many spaceflight experiments including those that focused on seedlings grown in the dark (Kwon et al., 2015; Kruse et al., 2020). The investigators who conducted the EMCS experiments SG2 and SG3, (Vandenbrink et al., Herranz et al., and Villacampa et al.) reported that genes involved in photosynthesis were enriched in both µ *g* upregulated and µ *g* downregulated datasets. We did not detect photosynthesis related genes upregulated in µ *g* in either PS or PRR, perhaps due to the differences in the lighting regime compared with the SG series of experiments. However, we do see enrichment for genes involved in photosynthesis and plastid function downregulated in µ *g*. Plastid localization was an enriched GO term for cellular localization accounting for approximately 300 genes in the µ *g* downregulated category of our dataset. Genes encoding for subunits of the NADPH complex, LHCA and photosystem I and II core were downregulated in µ *g* in both PS and PRR. We also found that several of the µ *g* downregulated miRNA targets were associated with plastids. Since many of these spaceflight studies have focused on young Arabidopsis seedlings, it is not clear if the altered regulation of genes associated with photosynthesis and chloroplast function would persist in fully grown plants. Whether such adjustments continue to maturity and how they may impact the photosynthetic capacity of plants will need to be evaluated in future spaceflight experiments.

#### 4.1.3 HY5 target genes

Genes differentially expressed in µ *g* in both PS and PRR were significantly enriched for HY5 target genes. HY5 is a central regulator of photomorphogenesis and may directly or indirectly affect the expression of approximately one third of the Arabidopsis genome (Lee et al., 2007; Gangappa and Botto, 2016). HY5 is involved in integrating light and hormonal signals to regulate developmental processes in plants. In addition to a major role in light mediated signaling, HY5 is implicated in nutrient uptake and utilization, biosynthesis of secondary metabolites and response to fluctuations in temperature (Xiao et al., 2021). HY5 targets were significantly enriched in the genes upregulated in µ *g* (hypergeometric test, *p*=2.45e-11). The µ *g* upregulated HY5 targets were associated with organ development, light responses and auxin transport. We also found a significant enrichment of HY5 targets in the genes downregulated in µ *g* (hypergeometric test, *p*=1.698e-120). Many of the downregulated HY5 targets were involved in abiotic and biotic stress responses.

#### 4.1.4 Gravitropism related genes

Curiously, we found some gravitropism related genes to be upregulated in µ *g*. Among these, the TF Shoot Gravitropism 5 (*SGR5*) was upregulated in µ *g* at both Day 4 and Day 6. SGR5 was first described as being important for the gravitropic response of Arabidopsis inflorescence stems (Yamauchi et al., 1997). Further studies showed that SGR5 is localized in the shoot endodermis and that amyloplast sedimentation in the shoot endodermis is slower in the *sgr5* mutant (Morita et al., 2006; Tanimoto et al., 2008). SGR5 is also known as Indeterminate Domain 15 (IDD15). The IDD TFs IDD14, IDD15 and IDD16 have been shown to work cooperatively to regulate auxin transport in the shoot and play a role in aerial tissue development and gravitropic responses (Cui et al., 2013). We found *IDD14* to be upregulated in µ *g*, as well as another gene involved in regulating gravitropic response – *LAZY1*. *LAZY1* is expressed primarily in shoots of young seedlings and the early stages of the hypocotyl gravitropism were affected in a *lazy1* mutant (Yoshihara and Spalding, 2017). The LAZY proteins are thought to act early in the gravity signaling cascade by transmitting positional information on amyloplast sedimentation upstream of auxin redistribution (Nishimura et al., 2023). The upregulation of these genes under µ *g* maybe related to their roles in regulating auxin flow; a closer look at their localization and dynamics under microgravity would be informative.

### 4.2 The Influence of developmental stage on observed transcriptional responses

The differences in gene expression between µ *g* and 1 *g* at Day 4 and Day 6 in PRR, revealed that many more genes were responsive to µ *g* at Day 6 than at Day 4. Since the Day 4 and Day 6 samples for each condition were obtained from the same rotor and collected at the same time, these differences suggest that the developmental stage of the seedlings can have a profound effect on observed transcriptional responses. The variation between Day 4 and Day 6 samples grown in 1 *g* and µ *g*, was also evident in the PCA plot where principal component 1, (which captured 53% of the variation between samples), clearly separated the Day 4 and Day 6 samples at 1 *g*; however, this separation was not observed in samples grown under the µ *g* condition. This indicates that differences in gene expression between the Day 4 and Day 6 samples in 1 *g* are either masked or not present in µ *g*. A possible explanation for this observation is that there are differences between 1 *g* and µ *g* grown seedlings in their developmental progression.

To determine if there were delays in germination between 1 *g* and µ *g* that could result in altered development we examined germination time by interrogating the sequential series of images obtained throughout the PRR experiment. We found no difference in the timing of the first occurrence of radicle emergence between µ *g* and 1 *g* seedlings. We conclude that there was no delay in germination under µ *g*; additionally, growth at Day 6 (**Figure 3**) was similar between µ *g* and 1 *g* conditions. Although we did not see gross differences in growth, given the short timeframe of this experiment, we cannot rule out that there may be subtle changes which could become more pronounced as the plants mature. Longer duration experiments with a finer resolution of time points would be required to fully characterize and understand the regulatory underpinnings for these differences.

### 4.3 Lessons learned for future spaceflight experiments

#### 4.3.1 Long term plant experiments

The PS and PRR experiments were focused on a narrow window of the Arabidopsis life cycle; the TROPI seed cassettes and EC arrangement only supported short duration experiments. Therefore we cannot extrapolate or assume that the transcriptional response to microgravity seen in these experiments would be universally applicable to long duration plant experiments on the ISS. Furthermore the seed cassettes provide a controlled optimal environment for seedling growth with sufficient lighting, moisture and humidity without inherent spaceflight associated limitations of water delivery that are manifest in larger plant growth habitats. Long term experiments where plants are grown from seed to maturity are needed to characterize critical stages in plant development. This approach will lead to a better understanding of the physiology of plant adaptations to the space environment.

#### 4.3.2 Multiple bioreplicates

Both PS and PRR included two experimental runs or bioreplicates. Although the replicates for each spaceflight were carried out back to back in the same hardware, and from the same flight builds, we detected run to run variation in responses. Run to run variation may be caused by other spaceflight related conditions that could differ on a day to day basis on the ISS. While this disparity did not overshadow the major transcriptional changes that were conserved between the PS and PRR experiments, it could be misleading if results from only a single experiment are considered. This underscores the importance of being able to conduct replicate spaceflight experiments.

#### 4.3.3 Combined OMIC approaches

We acknowledge that single-time-point studies are snapshots of “steady-state” transcriptional profiles which, on their own, are not sufficient to capture the full range and continuum of regulation. However, single-time-point transcriptional studies are valuable in that they can help identify key transcriptional regulators and they offer the first signs of pathways which may be altered. The inclusion of sRNA profiling in combination with RNA-Seq can provide clues on an additional layer of post transcriptional regulation and upcoming spaceflight experiments are likely to incorporate both these forms of analyses. While the proteome may be a more direct readout of response, spaceflight experiments frequently have insufficient amounts of tissue for large scale proteomic studies; additional hurdles include the cost and the paucity of well annotated reference protein databases. Only a few flight experiments to date, have taken a proteomic approach (Ferl et al., 2014; Mazars et al., 2014; Zhang et al., 2015; Kruse et al., 2020), and fewer still have been able to include both proteomic and transcriptomic analyses (Kruse et al., 2020). Moving forward it will be important to employ multiple OMICs approaches to study a spaceflight experiment. The larger plant habitats on the ISS such as Veggie and the Advanced Plant Habitat (APH) can better support these endeavors. Ultimately, the biochemical and metabolic status of the plants will be indicative of their adaptations to their new environment.

## Data availability statement

The datasets presented in this study can be found in online repositories. The names of the repository/repositories and accession number(s) can be found below: NASA GeneLab data repository, OSD-223 and OSD-437.

## Author contributions

EL: Investigation, Writing – original draft, Writing – review & editing, Methodology, Visualization, Software. JS: Methodology, Writing – original draft, Data curation, Formal analysis. CD: Formal analysis, Methodology, Writing – original draft, Writing – review & editing, Supervision. IP: Writing – original draft, Writing – review & editing, Conceptualization, Funding acquisition, Investigation, Project administration, Supervision, Data curation.
