# Supplementary figures and images for: Conserved plant transcriptional responses to microgravity from two consecutive spaceflight experiments
Source: Front Plant Sci. 2024 Jan 8;14:1308713. doi: 10.3389/fpls.2023.1308713 (PMC10800490; doi:10.3389/fpls.2023.1308713)

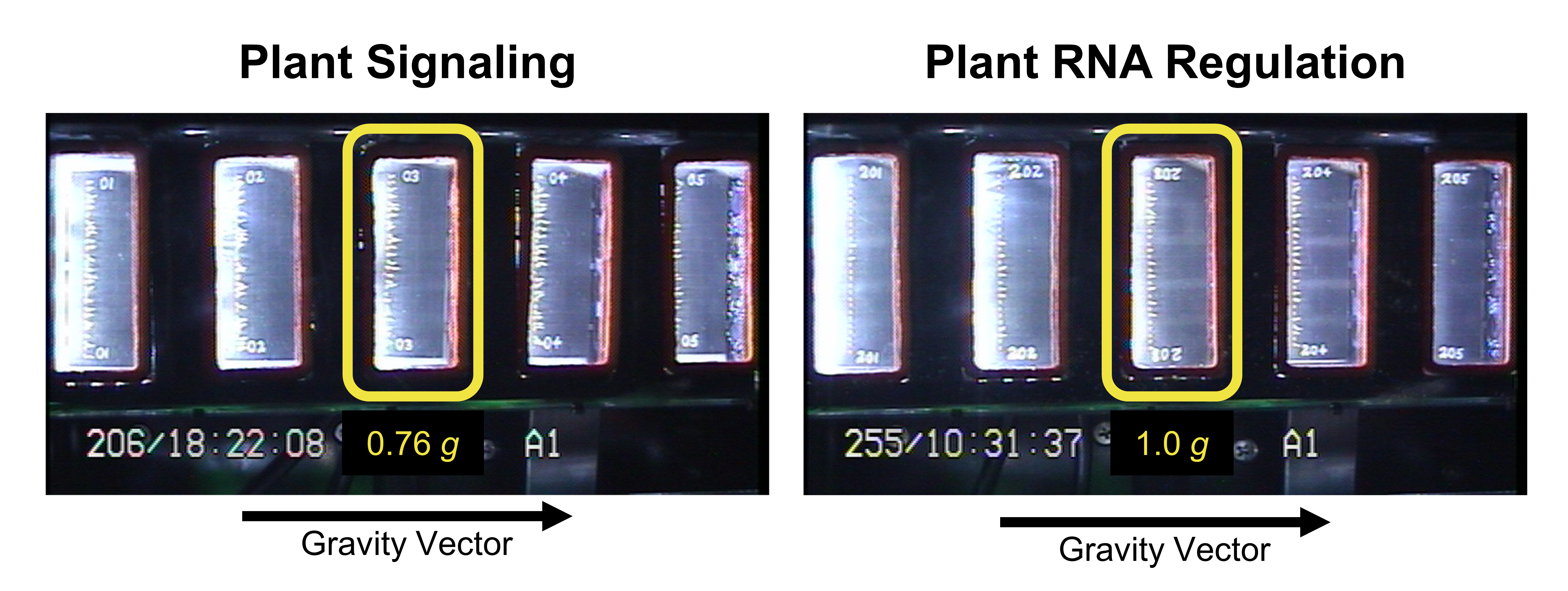

Supplement: Supplementary file 1 [file Image_1.tif]

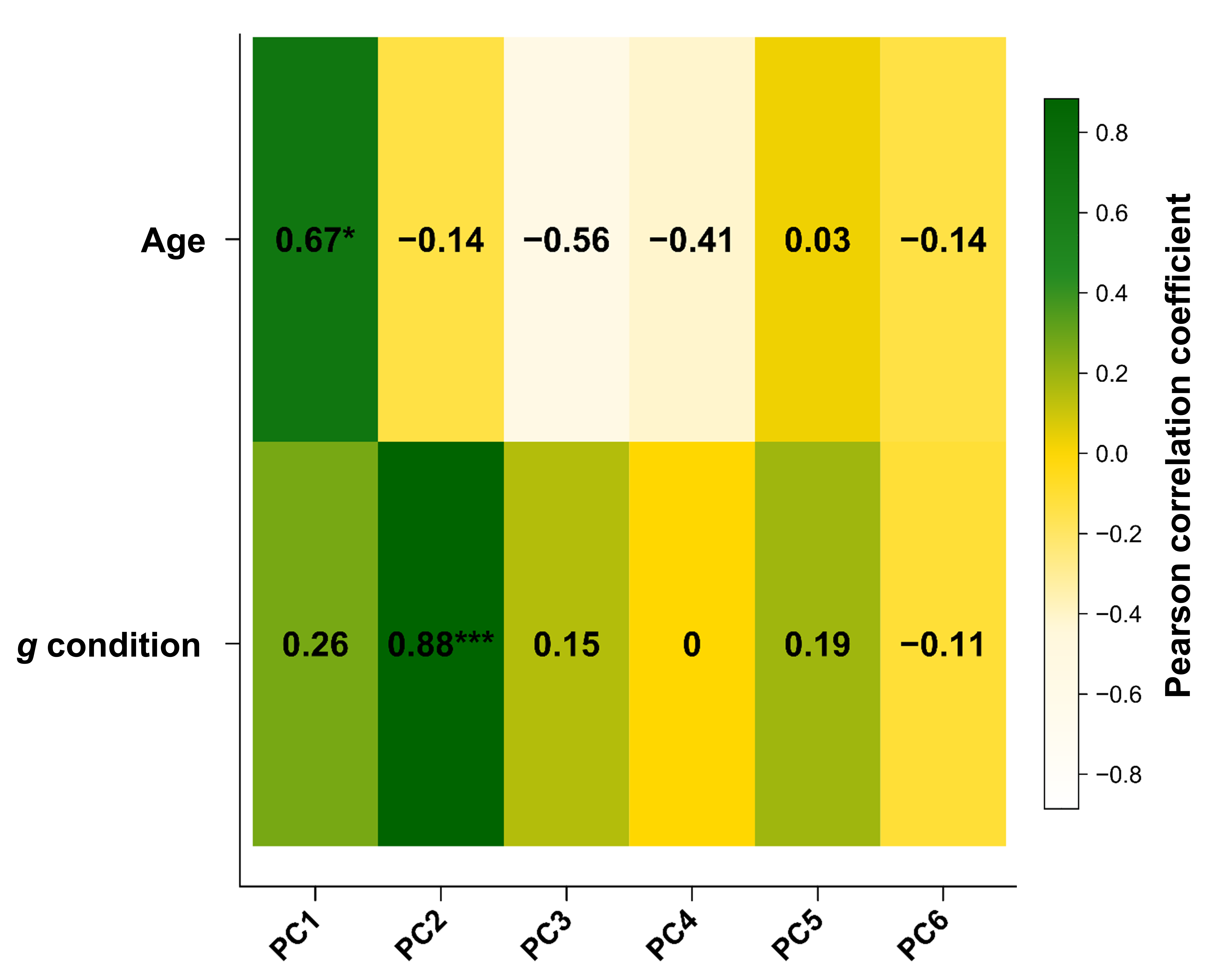

Supplement: Supplementary file 2 [file Image_2.tif]
