# Supplementary material for: Conserved plant transcriptional responses to microgravity from two consecutive spaceflight experiments
Source: Front Plant Sci. 2024 Jan 8;14:1308713. doi: 10.3389/fpls.2023.1308713 (PMC10800490; doi:10.3389/fpls.2023.1308713)
Supplement: Supplementary file 5 [file Table_2.docx]

**Legends for Supplementary Figures:**

**Supplementary Figure S1: Overview of an EC from the “1g” rotor from PS and PRR.**  The figure shows the arrangement of the 5 seed cassettes in each EC and the direction and g gradient across the EC. The 3^rd^ position cassette used for these studies is outlined in yellow.

**Supplementary Figure S2: Day 4 vs Day 6 Significance Correlation.** The correlation between the top six principal components and the variables of Age and Gravity level was evaluated. The correlation between the principal component and each variable was visualized and evaluated using the eigencorplot function in the PCATools R package. The direction of the correlation is shown by the color, green is positively correlated and yellow is more negatively correlated. The Pearson’s r value is shown in each cell and the significance level is indicated by an * for p-value < 0.05, and *** for p-value < 0.001.

**Figure S3:**Representative time series of raw image captures downlinked from PS and PRR 1 *g* and μ *g*experimental runs. Seedlings germinated with similar timing and growth across treatments in each experiment. Geometric reference is given by the grey gridded membranes to which seedlings are mounted; each gridded square comprises a dimension of 3.1 mm².
